# THz-circuits driven by photo-thermoelectric, gate-tunable graphene-junctions

**DOI:** 10.1038/srep35654

**Published:** 2016-10-20

**Authors:** Andreas Brenneis, Felix Schade, Simon Drieschner, Florian Heimbach, Helmut Karl, Jose A. Garrido, Alexander W. Holleitner

**Affiliations:** 1 Walter Schottky Institut and Physics Department, Technical University Munich, Am Coulombwall 4a, 85748 Garching, Germany; 2 Nanosystems Initiative Munich (NIM), Schellingstr. 4, 80799 Munich, Germany; 3 Lehrstuhl für Physik funktionaler Schichtsysteme, Physics Department, Technical University of Munich, D-85748 Garching, Germany; 4 Institute of Physics, University of Augsburg, 86135 Augsburg, Germany

## Abstract

For future on-chip communication schemes, it is essential to integrate nanoscale materials with an ultrafast optoelectronic functionality into high-frequency circuits. The atomically thin graphene has been widely demonstrated to be suitable for photovoltaic and optoelectronic devices because of its broadband optical absorption and its high electron mobility. Moreover, the ultrafast relaxation of photogenerated charge carriers has been verified in graphene. Here, we show that dual-gated graphene junctions can be functional parts of THz-circuits. As the underlying optoelectronic process, we exploit ultrafast photo-thermoelectric currents. We describe an immediate photo-thermoelectric current of the unbiased device following a femtosecond laser excitation. For a picosecond time-scale after the optical excitation, an additional photo-thermoelectric contribution shows up, which exhibits the fingerprint of a spatially inverted temperature profile. The latter can be understood by the different time-constants and thermal coupling mechanisms of the electron and phonon baths within graphene to the substrate and the metal contacts. The interplay of the processes gives rise to ultrafast electromagnetic transients in high-frequency circuits, and it is equally important for a fundamental understanding of graphene-based ultrafast photodetectors and switches.

Graphene is a gapless broadband absorber, which allows to detect wavelength-multiplexed signals in the frequency range from THz to UV-Vis-radiation^1^. In particular, free-charge carrier excitations can be exploited for THz signal propagation^2^, whereas interband excitations, like photogenerated electron-hole pairs, can drive ultrafast currents because of a femtosecond relaxation of photogenerated charge-carriers. A corresponding ultrafast photovoltaic current is driven by built-in electric fields, for instance, at metal-graphene junctions^3^,^4^,^5^,^6^,^7^ and by an externally applied bias voltage^8^. An alternative route to harvest and exploit the energy of incident photons are so-called photo-thermoelectric currents, which have been reported for graphene *p-n* junctions^9^,^10^,^11^,^12^,^13^, junctions made from mono- and bilayer graphene^14^, and graphene junctions with asymmetric metal contacts^15^. The thermopower differs on the two adjacent sides of these junctions, and the optical excitation generates the necessary heat to drive the thermoelectric current. Graphene is particularly suited to host thermoelectric currents, because the electron-electron scattering is the dominant ultrafast decay channel for a low irradiation intensity^16^. In turn, a hot electron distribution can quickly form after the optical excitation with only a small dissipation to the crystal lattice^13^,^17^,^18^,^19^. Hereby, non-equilibrium charge carrier temperatures of 10^3^–10^4^ K can be achieved^16^,^20^. Such an elevated temperature in combination with the large thermopower of graphene (≈100 μV/K) gives rise to a significant photo-thermoelectric response. As it is shown here, the latter can be used for triggering ultrafast signals in high-frequency circuits.

We exploit an on-chip pump-probe photocurrent spectroscopy based on THz-waveguides and Auston-switches with a sub-picosecond time-resolution^7^,^21^,^22^,^23^. Our technique can detect photocurrents that are linear with respect to the laser power^13^,^16^, and moreover, we detect signals which trigger electromagnetic transients in the THz circuits^21^,^24^,^25^. We can distinguish two ultrafast contributions in the graphene-junctions. On the one hand, an immediate response occurs during the pulsed excitation. This immediate response exhibits a six-fold sign-change as a function of both gate voltages of the dual-gated junction. This is a clear finger-print of a photo-thermoelectric current^9^,^11^,^19^. On the other hand, we observe a decaying photocurrent contribution on the time-scale of a few picoseconds. We can numerically compute this decaying response by a thermoelectric current which is driven by an inverted temperature profile within the graphene device. The non-equilibrium temperature inversion occurs because the electron and phonon baths of the graphene couple differently to the metal contacts and to the underlying substrate of the device. Our results show that the heat loss to the immediate environment of the graphene plays an important role in optimizing the energy conversion efficiency in graphene. The insights into the non-equilibrium carrier dynamics may proof essential for future ultrafast optoelectronic switches and for the fundamental understanding of photovoltaic devices and photodetectors. In particular, our work experimentally proves that photo-thermoelectric currents can be used for an ultrafast optoelectronic signal-conversion in graphene as a functional part of high-frequency circuits^26^,^27^. We show how dual-gated graphene junctions can be designed and fabricated such that they drive on-chip THz-circuits. Since graphene junctions can act as very efficient far-field THz-detectors detectors^15^, our work on gate-tunable, photo-thermoelectric graphene-junctions suggest that they can act as off/on-chip interconnects in the THz-regime.

## Results

### Sample Fabrication

**Figure 1:**
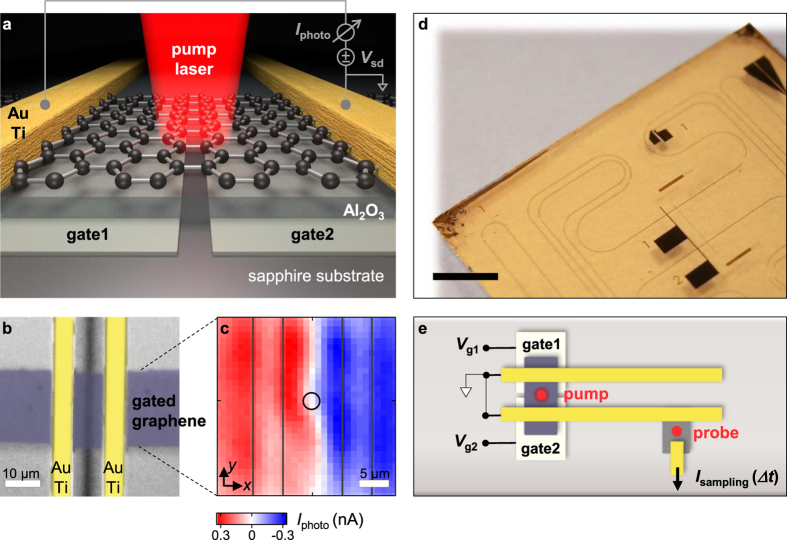
Gated graphene-junction integrated in a THz circuit. (**a**) Sketch of a dual-gated graphene junction with two bottom gates, named gate1 and gate2, on top of a sapphire substrate and an insulating Al_2_O_3_-layer in-between. The graphene is contacted by two Ti/Au-striplines. A pulsed laser generates charge carriers in the graphene. (**b**) Microscope image of a corresponding graphene sheet (highlighted in blue) and the striplines (yellow) on top of two gates. The lateral gap between both gates is ≈2 μm (dark region in the center). (**c**) Spatially resolved and time-integrated photocurrent *I*_photo_ of the graphene sheet shown in Fig. 1b as a function of the coordinates *x* and *y*. For *x* = *y* = 0 μm, the sample is excited at the positon of the circle. The vertical contour lines depict the positions of the striplines. (**d**) Image of the overall THz-stripline circuit on a sapphire chip. Scale bar, 2 mm. (**e**) Sketch of the on-chip pump/probe scheme. A pump pulse excites the gated graphene-junction, while the probe pulse triggers an Auston-switch. The latter is an ultrafast photoswitch and it reads out the electromagnetic transients of the striplines. As a function of the time-delay *Δt*, the corresponding current *I*_sampling_ gives insights into the electromagnetic signals running along the high-frequency striplines and hereby, into the temporal optoelectronic dynamics of the graphene-junction.

Figure 1a sketches the sample layout of the dual-gated graphene junction. The circuit provides two gates labeled gate1 and gate2. Applying corresponding gate voltages *V*_g1_ and *V*_g2_ controls the Fermi levels and thus the thermopower in the graphene on each side of the junction. The graphene is electrically contacted by metal strips made from Ti/Au (10 nm/250 nm), which allow to apply a bias voltage *V*_sd_. First, two adjacent 10 nm thick Pt gates are lithographically defined on a sapphire substrate. Then, a 30 nm thick, ALD-grown (atomic layer deposition) Al_2_O_3_-layer isolates the gates from the graphene. Subsequently, CVD-grown (chemical vapor deposition) graphene is mechanically transferred to the sample^28^. The graphene region of interest is 25 μm wide and meanwhile protected with a resist, while the remaining graphene is removed by utilizing an oxygen plasma. In a final lithography step, the protective resist is removed by a solvent, and the striplines are lithographically defined on top of the graphene. Figure 1b shows an optical microscope image of the device with the graphene (striplines) being highlighted in blue (yellow). The gap between the two underlying Pt-gates is clearly visible as dark contrast. Moreover, the Ti/Au-contacts form coplanar high-frequency striplines with a total length of ≈44 mm, a width of 5 μm, and a separation of 10 μm. The striplines are needed to measure the time-resolved photocurrent *I*_sampling_^7^,^21^.

### Scanning photocurrent microscopy

For the excitation of the graphene, we use a laser system that emits laser pulses with a 30 fs pulse duration at a repetition rate of 80 MHz in the spectral range from 900 to 1500 nm (cf. Supplementary Figure S1). The laser beam is focused with a 15x reflective objective. The laser spot on the surface of the graphene has a full width half maximum (FWHM) of ≈5.5 μm. All presented experiments are performed with an excitation power of 1–2 mW at room temperature and in a vacuum chamber. First, the time-integrated photocurrent *I*_photo_ is measured with the help of a current-voltage amplifier while the sample is scanned laterally by the help of piezoelectric positioners. Figure 1c depicts such a photocurrent map of *I*_photo_ at *V*_sd_ = *V*_g1_ = *V*_g2_ = 0V. For these experimental parameters, *I*_photo_ shows opposite signs on both sides of the junction, as it will be discussed below.

### THz-time domain photocurrent spectroscopy

For measuring the ultrafast optoelectronic dynamics of the graphene-based junction, we use an on-chip THz-time domain photocurrent spectroscopy of the stripline circuits (Fig. 1d)^7^,^21^,^22^,^23^, where an optical femtosecond pump laser excites the charge carriers in the graphene (Fig. 1e). This laser is the same as for the time-integrated photocurrent measurements (Fig. 1a). Since the contacts form striplines, the photocurrents give also rise to electromagnetic transients in the metal striplines with a bandwidth of up to 2 THz^21^,^22^. The transients run along the striplines, and they are detected on-chip by a time-delayed optical femtosecond probe pulse in combination with an Auston-switch (Fig. 1e)^21^. We use ion-implanted amorphous silicon for this ultrafast photodetector with a time-resolution of about 1 ps^22^,^23^. The probe pulse has a photon energy of 1.59 eV and a temporal width of 100 fs. The current *I*_sampling_ across the Auston-switch samples the electromagnetic transients on the striplines as a function of the time-delay *Δt* between the two laser pulses, and it is directly proportional to the ultrafast photocurrents in the graphene. Hereby, the photocurrents in the graphene can be measured with a picosecond time-resolution^7^,^21^.

### Resistance properties of the graphene junction

**Figure 2:**
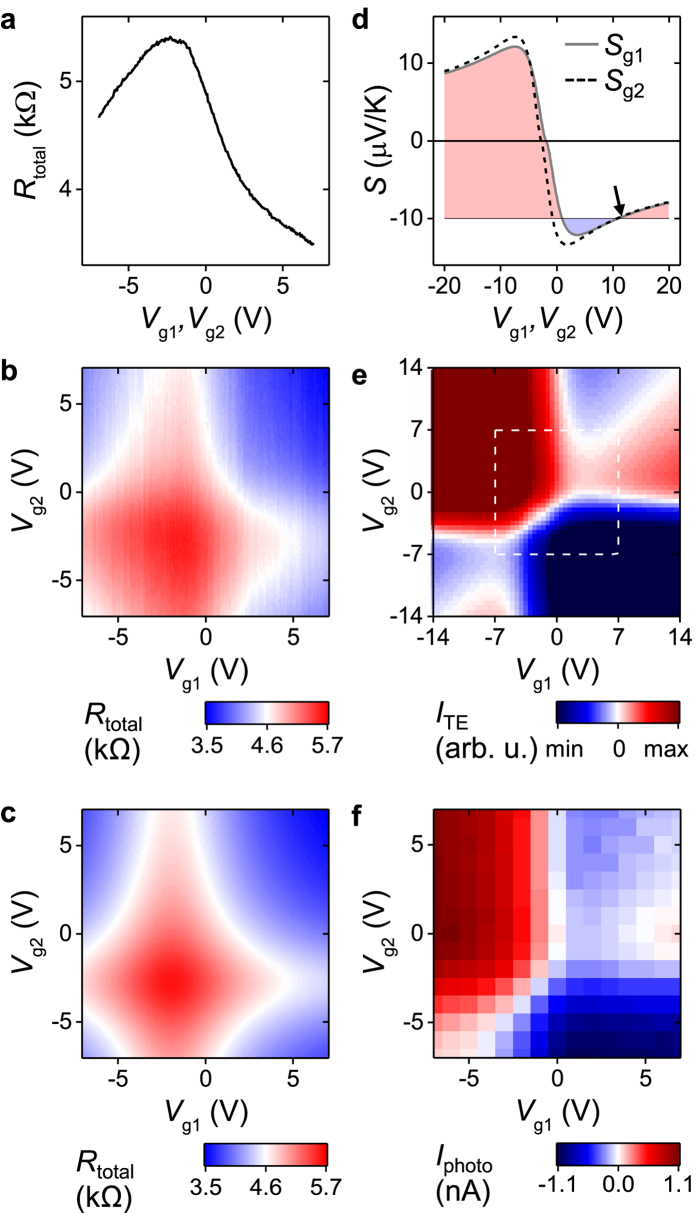
Electronic and photo-thermoelectric properties of the graphene-junction. (**a**) Two-terminal resistance *R*_total_ of the dual-gated graphene junction, when the two gate voltages *V*_g1_ and *V*_g2_ are swept simultaneously. The maximum gives the charge neutrality point of the graphene sheet. (**b**) Two-dimensional resistance plot *R*_total_ = *R*_total_(*V*_g1_,*V*_g2_), when the gates are varied individually. (**c**) Two-dimensional fit of *R*_total_ according to Eq. (2). See text for details. (**d**) Seebeck coefficient *S*_g1_ (*S*_g2_) vs. *V*_g1_ (*V*_g2_) calculated from the fit parameters of *R*_total_ according to Eq. (5). (e) Calculated thermoelectric current vs. *V*_g1_ (*V*_g2_) for the doping and mobility parameters from (c) according to Eq. (3) and *I*_TE_ = *V*_TE_/*R*_total_. (**f**) Experimental time-integrated photocurrent *I*_photo_ vs. *V*_g1_ (*V*_g2_) at *x* = *y* = 0 (circle in Fig. 1c).

The two-terminal resistance *R*_total_ is measured with a current-voltage amplifier to gain insights into the electronic properties of the dual-gated graphene junction. Figure 2a shows the experimental dependence of *R*_total_, when both voltages of gate1 and gate2 are varied simultaneously. We find the charge neutrality point of the graphene for negative gate voltages (*V*_d_ < 0*V*), which implies that it is intrinsically *n*-doped. Figure 2b shows *R*_total_, when *V*_g1_ and *V*_g2_ are varied independently. We apply the following model^29^ to approximate the charge carrier density in dependence of the applied gate voltage


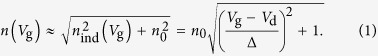


Here, *n*_0_ is the residual charge carrier density (*i.e.* the minimum sheet carrier concentration), *n*_ind_ the gate-induced charge carrier density, *n* the total charge carrier density. The term Δ is defined as 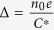, with C^*^ the specific capacitance 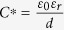 of the gate and the graphene-sheet^29^ (with *ε*_*r*_ = 6.0 the relative permittivity and *d* = 30 nm the thickness of the Al_2_O_3_ layer). The data in Fig. 2b can be fitted by


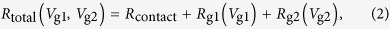


The expression *R*_contact_ accounts for the constant resistance of the metal striplines and the ungated graphene region. *R*_*gi*_ represents the gate voltage dependent resistance of the two (*i *= 1,2) gated graphene sections, respectively. This resistance is related to the conductivity by 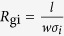, where *l* = 4 *μ*m and *w* = 25 *μ*m are the length and width of the gated graphene sections, respectively. The conductivity *σ*_*i*_(*V*_*gi*_) can be tuned by the gate voltage *V*_*gi*_ and is calculated from 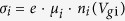 using Eq. (1). Our fit gives *R*_contact_ = 2.57 kΩ and mobilities of *μ*_1_ = 157 cm^2^V^−1^s^−1^ and *μ*_2_ = 278 cm^2^V^−1^s^−1^. The charge neutrality point is reached at *V*_d1_ = −1.92 V and *V*_d2_ = −2.77 V. The fit further reveals Δ_1_ = 3.20 V and Δ_2_ = 2.62 V. Figure 2c shows the resulting fit (cf. Supplementary Figures S2 and S3).

### Photo-thermoelectric properties

The photo-thermoelectric effect^9^,^10^,^11^,^19^ and the photovoltaic effect^3^,^4^,^6^,^7^,^8^ are considered as the two distinctive photocurrent generation mechanisms in graphene on short time-scales. The photovoltaic effect relies on a drift current, where photogenerated charge carriers are separated by an electric field. In turn, the photovoltaic current monotonically depends on the difference of the Fermi levels of both sides of a dual-gated graphene junction (cf. Supplementary Figure S4). In the case of a thermoelectric generation mechanism, the photocurrent is a non-monotonic function. In particular, the thermoelectric voltage *V*_TE_ can be written as


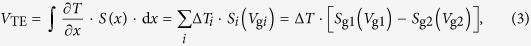


with Δ*T*_*i*_ the change in electron temperature for a segment with constant thermopower *S*_*i*_. We define the *x*-axis as in Fig. 1c. The expression can be simplified to the last term of Eq. (3) for a symmetric heat profile with amplitude Δ*T* and for constant thermopowers *S*_g1_ and *S*_g2_ for the two areas on top of gate1 and gate2. We note that a symmetric heat profile is introduced by the laser pulse with the given FWHM of ≈5.5 μm (Fig. 1a). The thermopower or so-called Seebeck coefficient can be related to the electric properties of the device following the Mott formula^30^.


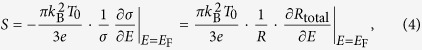


with *E*_F_ the Fermi-energy, *k*_B_ the Boltzmann constant, *e* the elementary charge, *σ* the electric conductivity, and *T*_0_ = 300 K the equilibrium electron temperature. With the definitions from Eq. (1) and 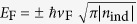, Eq. (4) can be rewritten for each gate voltage


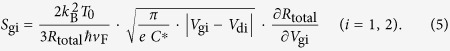


Equation (5) is used to calculate the thermopower (Seebeck coefficients) *S*_*gi*_ = *S*_*gi*_(*V*_*gi*_) from the fit of *R*_total_ = *R*_total_(*V*_g1_,*V*_g2_) in Fig. 2c. Figure 2d allows to intuitively understand the non-monotonic behavior of the thermopower as a function of *V*_g1_ and *V*_g2_: for a given gate voltage *V*_g2_ and Seebeck coefficients *S*_g2_ (black arrow in Fig. 2d), there are two possible gate voltages *V*_g1_, which result in a coincident Seebeck coefficient *S*_g1_ = *S*_g2_. In turn, the thermoelectric current will show two crossing points as a function of *V*_g1_ and *V*_g2_ according to the right-hand-side of Eq. (3). The resulting six-fold sign change is therefore a characteristic, non-monotonic fingerprint of a thermoelectric current (Fig. 2e)^9^,^11^,^19^. In the calculus, the gate voltages are varied from −14 to +14 V. Experimentally, the breakdown voltage of the Al_2_O_3_-layer limits the maximum sweeping range to ± 7 V (dashed square in Fig. 2e). Figure 2f presents the corresponding time-integrated photocurrent *I*_photo_ of the dual-gated junction as a function of *V*_g1_ and *V*_g2_, when the exciting laser is focused onto the center of the graphene junction (circle in Fig. 1c). Given the limited gate sweeping range, we clearly observe the sign change as expected from the pattern of the calculated thermoelectric current in Fig. 2e.

### Ultrafast currents in the graphene-junction

**Figure 3:**
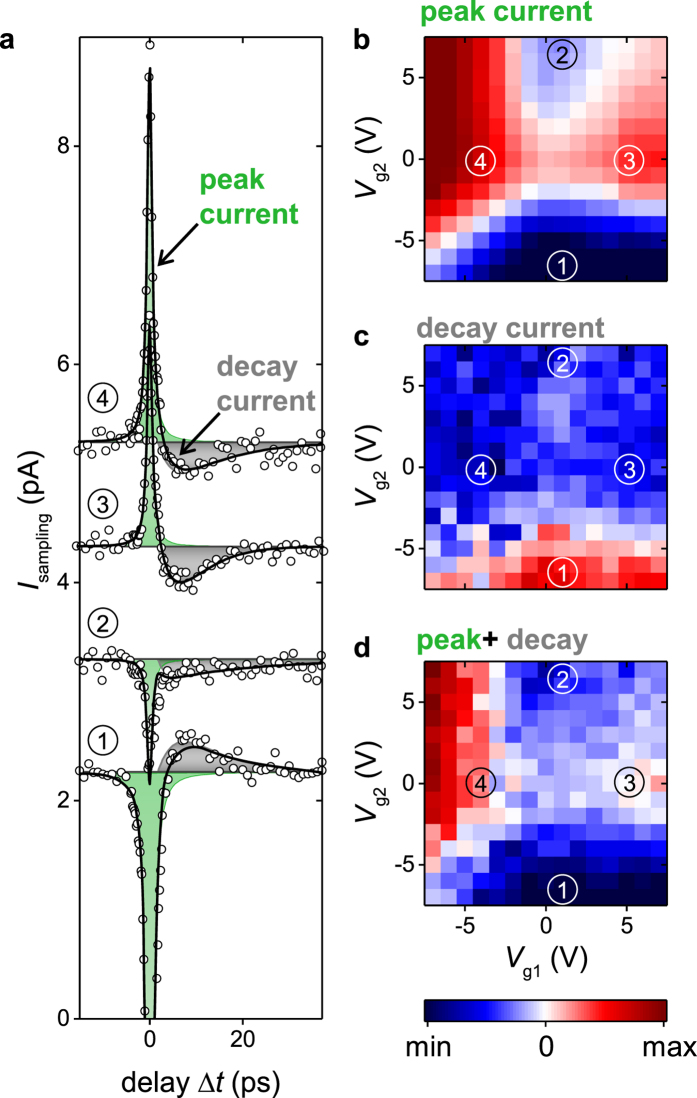
Ultrafast photo-thermoelectric currents in the graphene-junction measured by the THz-circuit. (**a**) Time-resolved photocurrent *I*_sampling_(*Δt*) for four different gate voltages at the position *x* = *y* = 0 (circle in Fig. 1c) with a distinctive ‘peak current’ (green area) and a ‘decay current’ (gray area). (**b**) Area of the peak current vs. *V*_g1_ and *V*_g2_ with the four gate voltage settings 1, 2, 3, and 4 of (**a**,**c**) Area of the decay current vs. *V*_g1_ and *V*_g2_. (**d**) Sum of the areas in figures (**b**,**c**).

In the following, we discuss the temporal dynamics of the non-equilibrium photocurrents in the graphene junction as detected by the THz stripline circuit. Figure 3a depicts the time-resolved photocurrent *I*_sampling_(*Δt*) for different gate voltages *V*_g1_ and *V*_g2_. The voltage settings are defined by the indices in Fig. 3b. The signal can be described by a ‘peak current’ as an immediate response (green area in Fig. 3a), that occurs when the delay Δ*t* between pump and probe pulse is zero. For a longer time-delay, we observe a rising and decaying behavior of *I*_sampling_ (gray area). We label the latter response as ‘decay current’. For each gate voltage setting, we fit *I*_sampling_(*Δt*) with a constant offset, a Lorentzian (green area), and an exponentially rising and decaying function 
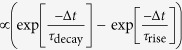
 (gray area). The latter is a phenomenological function to describe the form of the decay current with rise and decay times *τ*_decay_ and *τ*_rise_^21^. They will be discussed below. The temporal width of the Lorentzian is 1.28 ± 0.18 ps, which coincides with the bandwidth limitation of the present stripline circuit^31^. The bandwidth is limited by the dispersion and attenuation of the striplines. The dependences of the peak and decay currents on the gate voltages are depicted in Fig. 3b,c, respectively. The peak current exhibits a manifold sign change, which is in agreement with a thermoelectric current within the limited gate voltage range (compare to the highlighted square in Fig. 2e). The decay current rather depends on *V*_g2_ than on *V*_g1_ for this laser position. We further note that the peak current and the decay current can have different polarities at certain gate voltages (e.g. setting 1, 3, and 4 in Fig. 3a). At the given signal-to-noise-ratio, however, the sum of the peak and decay current (Fig. 3d) mimics the time-integrated signal *I*_photo_ (Fig. 2f) to a better extend than the peak current alone (Fig. 3b). This finding is consistent with the fact that the time-integrated signal comprises the sum of all ultrafast currents.

## Discussion

We note that we read-out the ultrafast photocurrent results with a picosecond time-resolution in an on-chip manner only because of the split-gate design as sketched in Fig. 1a. For a global back gate which spans below one stripline to the other, the high-frequency components of the striplines are damped-out. A design guideline for the integration of gate-tunable graphene-junctions into THz-circuits is given in the Supplementary Note (Supplementary Figures S5–S10).

In order to explain the measured ultrafast peak and the decay currents in Fig. 3, we consider the ultrafast charge carrier relaxation dynamics. After the pulsed excitation, the charge carriers relax due to electron-electron scattering on a femtosecond time-scale, and the carrier ensemble can quickly be described by a hot Fermi-Dirac distribution^13^,^16^,^19^,^32^,^33^,^34^,^35^,^36^. The electron and phonon bath temperatures equilibrate on a picosecond time-scale^13^,^17^,^18^,^19^ due to optical phonon scattering^37^,^38^, surface polar phonon scattering^39^,^40^, and supercollision scattering^41^,^42^,^43^. From the striking similarity of the expected pattern of the thermoelectric current in Fig. 2e and the measured dependence of the ‘peak current’ in Fig. 3b, we can conclude that this immediate photocurrent is dominated by an ultrafast thermoelectric current at the junction. The necessary non-equilibrium electron temperature can be assumed to be as high as 10^4^ K^16^,^20^. We find that it is important to excite at the center of the dual-gated junction such that a symmetric heat profile is impinged by the Gaussian laser profile with its given FWHM (sketch in Fig. 1a and circle in Fig. 1c). Hereby, the balanced temperature profile (|Δ*T*_1_| = |Δ*T*_2_| = |Δ*T*) and the difference in the thermopowers on both sides of the graphene give rise to a thermoelectric voltage *V*_TE_ = (*S*_g1_−*S*_g2_) Δ*T*, that drives a photocurrent as long as the temperature profile is present. We can further infer that the so-called photovoltaic effect plays only a negligible role. In particular, the photovoltaic effect would only depend on the difference of both Fermi levels of dual-gated graphene junctions. In other words, its sign should change along the diagonals of Fig. 3b,c, which we do not observe (cf. Supplementary Information). We can also exclude a photo-Dember current^44^,^45^, because its amplitude should only depend on the difference of the electron- and hole-mobilities without a sign-change as in Fig. 3b,c. Instead, we demonstrate below that the observed decay current is of thermoelectric origin as well.

**Figure 4:**
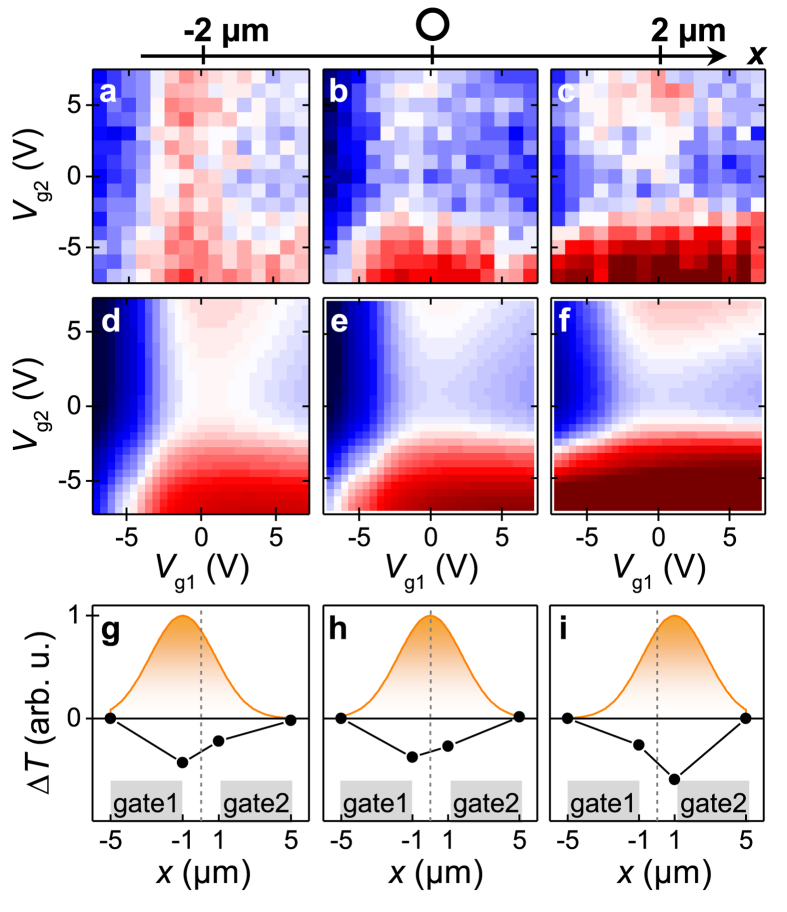
Heat profiling of the graphene-junction. (**a**–**c**): Area of the decay current (gray area in Fig. 3a) for three different positions of the laser spot (*x* = −2, 0, 2 μm, and *y* = −7 μm). (**d**–**f**): Thermoelectric current calculated for an inverted temperature profile defined in the figure (**g**,**h**), and (**i**). (**g**–**i**): Inverted temperature profile (black line) across the graphene junction for time-scales *Δt* > 1*ps*. The orange area depicts the Gaussian excitation intensity of the exiting laser pulse, i.e. the initial temperature profile at *Δt* = 0ps.

Figure 4a–c show the area of the decay current for a time-delay of 3.3 ps < *Δt* < 13.3 ps as a function of both gate voltages for three different excitation positions of the laser spot. The excitation position is varied in steps of 2 μm from an excitation position closer to gate1 (Fig. 4a), to one at the center between both gates (Fig. 4b), and to one closer to gate2 (Fig. 4c). We can numerically reproduce the corresponding patterns by Eq. (3) (Fig. 4d,e and f), if we consider a slight shift of the charge neutrality point and an inverted temperature profile (black dots in Fig. 4g,h and i). The shift of the charge neutrality point is induced by the position of the laser spot, because depending on the position, the static, optically induced background doping changes. For instance, the pattern of Fig. 4(a,c) with the laser spot on gate1 (gate2) shows a predominant dependence on *V*_g1_ (*V*_g2_). In other words, the decay current predominantly depends on the voltage of the gate at which the laser is focused. The numerical model (Fig. 4d–f and black data points in Fig. 4g–i) is used to qualitatively mimic both the opposite sign of the decay current compared to the peak current and the shift of the inverted temperature amplitude from gate1 to gate2. To explain the inverted temperature profiles, we need to consider the phonon bath of the graphene. It is heated on a picosecond time-scale by the mentioned electron-phonon scattering processes^17^,^18^,^19^,^37^,^38^,^39^,^40^,^41^,^42^,^43^. The heat will then be transferred to the environment of the graphene. In the case of the underlying Al_2_O_3_-layer and sapphire substrate, this will occur on a 1–10 ps time-scale^23^,^41^,^46^. On this time-scale, the temperature of the electron and phonon baths adopt the same values. In this sense, we interpret *τ*_decay_ and *τ*_rise_ of the decay current. For the metal striplines, the heat transfer occurs on a similar time-scale. However, the heat diffusion along the striplines is slower with a time-scale of up to 150–300 ps.7 Accordingly, the electron bath in the center of the dual-gated graphene junction efficiently cools-down faster than at the sections which are in vicinity to the metals. In turn, a thermoelectric current is generated with a corresponding longer time-scale at positions closer to one of the contacts, especially if the contacts are heated by the pump pulse^7^,^23^. On the technical side, the detected temporal width of the photo-thermoelectric response in our dual-gated graphene junction is 1.28 ± 0.18 ps. In our interpretation, the detected timescale is limited by the bandwidth limitation of the present stripline circuit^31^, because recently, it was demonstrated by a far-field THz-probe spectroscopy that hot electrons can exhibit a well-defined conductivity up to ~1.8 THz in graphene^16^. We finally note that both the ‘peak current’ and the ‘decay current’ are detected via our THz-time domain photocurrent spectroscopy. Therefore, both trigger ultrafast optoelectronic signals in the high-frequency circuit, and their polarity can be switched by the two gate voltages of the graphene-junction (Figs 3 and 4).

In conclusion, we show that the ultrafast photocurrent in a dual-gated graphene junction is dominated by the photo-thermoelectric effect. The Fermi energy of the two sides of the junctions is controlled by two adjacent back gates. Surprisingly, we find two distinct ultrafast thermoelectric contributions. A first immediate response occurs within the temporal resolution of our experiment, which is about one picosecond. An unambiguous dependence on the two gate voltages identifies this peak current as an ultrafast photo-thermoelectric current. In our interpretation, this current occurs on a sub-ps time-scale after the pulsed femtosecond laser excitation when the electrons form a hot Fermi Dirac distribution due to fast electron-electron scattering. A second ultrafast thermoelectric response is identified with a decaying constant of a couple of picosecond. We interpret the latter time-scale to represent the heat coupling of the electron and phonon baths to the environment of the graphene. We can numerically reproduce the second response by a thermoelectric current as long as the heat baths are out of equilibrium. The sum of the two time-resolved photocurrent contributions resembles the time-integrated photocurrent measured in the dual-gated graphene junction. The findings may proof essential for graphene-based photodetectors and switches integrated in future high-frequency circuits.

## Additional Information

**How to cite this article**: Brenneis, A. *et al.* THz-circuits driven by photo-thermoelectric, gate-tunable graphene-junctions. *Sci. Rep.* **6**, 35654; doi: 10.1038/srep35654 (2016).

## Supplementary Material

Supplementary Information

## References

[b1] Santos C. N. *et al.* Terahertz and mid-infrared reflectance of epitaxial graphene. Sci. Rep. 6, 24301 (2016). 27102827 10.1038/srep24301 PMC4840310

[b2] Williamson I. A. D., Hossein Mousavi S. & Wang Z. Extraordinary wavelength reduction in terahertz graphene-cladded photonic crystal slabs. Sci. Rep. 6, 25301 (2016). 27143314 10.1038/srep25301 PMC4855219

[b3] Peters E. C., Lee E. J. H., Burghard M. & Kern K. Gate dependent photocurrents at a graphene p-n junction. Applied Physics Letters 97, 193102 (2010).

[b4] Xia F. *et al.* Photocurrent Imaging and Efficient Photon Detection in a Graphene Transistor. Nano Lett. 9, 1039–1044 (2009). 19203207 10.1021/nl8033812

[b5] Xia F., Mueller T., Lin Y., Valdes-Garcia A. & Avouris P. Ultrafast graphene photodetector. Nature Nanotechnology 4, 839–843 (2009). 10.1038/nnano.2009.292 19893532

[b6] Park J., Ahn Y. H. & Ruiz-Vargas C. Imaging of Photocurrent Generation and Collection in Single-Layer Graphene. Nano Lett. 9, 1742–1746 (2009). 19326919 10.1021/nl8029493

[b7] Prechtel L. *et al.* Time-resolved ultrafast photocurrents and terahertz generation in freely suspended graphene. Nature Communications 3, 646 (2012). 10.1038/ncomms1656 22337124

[b8] Freitag M., Low T., Xia F. & Avouris P. Photoconductivity of biased graphene. Nature Photonics 7, 53–59 (2012).

[b9] Gabor N. M. *et al.* Hot Carrier–Assisted Intrinsic Photoresponse in Graphene. Science 334, 648–652 (2011). 21979935 10.1126/science.1211384

[b10] Lemme M. C. *et al.* Gate-Activated Photoresponse in a Graphene p–n Junction. Nano Lett. 11, 4134–4137 (2011). 21879753 10.1021/nl2019068

[b11] Song J. C. W., Rudner M. S., Marcus C. M. & Levitov L. S. Hot Carrier Transport and Photocurrent Response in Graphene. Nano Lett. 11, 4688–4692 (2011). 21936568 10.1021/nl202318u

[b12] Dun D. *et al.* Ultrafast hot-carrier-dominated photocurrent in graphene. Nature Nano 7, 114–118 (2012). 10.1038/nnano.2011.243 22245859

[b13] Tielrooij K. J. *et al.* Photoexcitation cascade and multiple hot-carrier generation in graphene. Nature Physics 9, 248–252 (2013).

[b14] Xu X., Gabor N. M., Alden J. S., van der Zande A. M. & McEuen P. L. Photo-Thermoelectric Effect at a Graphene Interface Junction. Nano Lett. 10, 562–566 (2010). 20038087 10.1021/nl903451y

[b15] Cai X. *et al.* Sensitive Room-Temperature Terahertz Detection via Photothermoelectric Effect in Graphene. Nature Nano. 9, 814 (2014). 10.1038/nnano.2014.182 25194945

[b16] Jensen S. A. *et al.* Competing Ultrafast Energy Relaxation Pathways in Photoexcited Graphene. Nano Lett., doi: 10.1021/nl502740g (2014). 25247639

[b17] Bistritzer R. & MacDonald A. H. Electronic Cooling in Graphene. Phys. Rev. Lett. 102, 206410 (2009). 19519053 10.1103/PhysRevLett.102.206410

[b18] Foster M. S. & Aleiner I. L. Slow imbalance relaxation and thermoelectric transport in graphene. Physical Review B 79 (2009).

[b19] Tielrooij K. J. *et al.* Generation of photovoltage in graphene on a femtosecond timescale through efficient carrier heating. Nat Nano 10, 437–443 (2015). 10.1038/nnano.2015.54 25867941

[b20] García de Abajo F. J. Graphene Plasmonics: Challenges and Opportunities. ACS Photonics 1, 135–152 (2014).

[b21] Auston D. H., Johnson A. M., Smith P. R. & Bean J. C. Picosecond optoelectronic detection, sampling, and correlation measurements in amorphous semiconductors. Applied Physics Letters 37, 371–373 (1980).

[b22] Kastl C., Karnetzky C., Karl H. & Holleitner A. W. Ultrafast helicity control of surface currents in topological insulators with near-unity fidelity. Nat Commun 6, 6617 (2015). 25808213 10.1038/ncomms7617 PMC4389261

[b23] Brenneis A. *et al.* Ultrafast electronic read-out of diamond NV centers coupled to graphene. Nat Nanotechnology 10, 135 (2015). 10.1038/nnano.2014.276 25437746

[b24] Hwang H. Y. *et al.* Nonlinear THz Conductivity Dynamics in P-Type CVD-Grown Graphene. J. Phys. Chem. B 117, 15819–15824 (2013). 24102144 10.1021/jp407548a

[b25] Frenzel A. J., Lui C. H., Shin Y. C., Kong J. & Gedik N. Semiconducting-to-Metallic Photoconductivity Crossover and Temperature-Dependent Drude Weight in Graphene. Phys. Rev. Lett. 113, 056602 (2014). 25126929 10.1103/PhysRevLett.113.056602

[b26] Zapata J. D. *et al.* Efficient graphene saturable absorbers on D-shaped optical fiber for ultrashort pulse generation. Sci. Rep. 6, 20644 (2016). 26856886 10.1038/srep20644 PMC4746624

[b27] Liu X. M. *et al.* Graphene-clad microfibre saturable absorber for ultrafast fibre lasers. Sci. Rep. 6, 26024 (2016). 27181419 10.1038/srep26024 PMC4867430

[b28] Blaschke B. M. *et al.* Flexible graphene transistors for recording cell action potentials. 2D Materials 3, 025007 (2016).

[b29] Kim S. *et al.* Realization of a high mobility dual-gated graphene field-effect transistor with Al2O3 dielectric. Applied Physics Letters 94, 062107 (2009).

[b30] Cutler M. & Mott N. F. Observation of Anderson Localization in an Electron Gas. Phys. Rev. 181, 1336–1340 (1969).

[b31] Prechtel L. *et al.* Time-Resolved Photoinduced Thermoelectric and Transport Currents in GaAs Nanowires. Nano Lett. 12, 2337–2341 (2012). 22494021 10.1021/nl300262j

[b32] Bao Q. & Loh K. P. Graphene Photonics, Plasmonics, and Broadband Optoelectronic Devices. ACS Nano 6, 3677–3694 (2012). 22512399 10.1021/nn300989g

[b33] Avouris P. & Freitag M. Graphene Photonics, Plasmonics, and Optoelectronics. IEEE Journal of Selected Topics in Quantum Electronics 20, 72–83 (2014).

[b34] George P. A. *et al.* Ultrafast Optical-Pump Terahertz-Probe Spectroscopy of the Carrier Relaxation and Recombination Dynamics in Epitaxial Graphene. Nano Lett. 8, 4248–4251 (2008). 19367881 10.1021/nl8019399

[b35] Kim R., Perebeinos V. & Avouris P. Relaxation of optically excited carriers in graphene. Phys. Rev. B 84, 075449 (2011).

[b36] Sun D. *et al.* Ultrafast Relaxation of Excited Dirac Fermions in Epitaxial Graphene Using Optical Differential Transmission Spectroscopy. Physical Review Letters 101 (2008). 10.1103/PhysRevLett.101.157402 18999638

[b37] Dawlaty J. M., Shivaraman S., Chandrashekhar M., Rana F. & Spencer M. G. Measurement of ultrafast carrier dynamics in epitaxial graphene. Applied Physics Letters 92, 042116 (2008). 10.1021/nl8019399 19367881

[b38] Wang H. *et al.* Ultrafast relaxation dynamics of hot optical phonons in graphene. Applied Physics Letters 96, 081917 (2010).

[b39] Freitag M., Low T. & Avouris P. Increased Responsivity of Suspended Graphene Photodetectors. Nano Lett. 13, 1644–1648 (2013). 23452264 10.1021/nl4001037

[b40] Low T., Perebeinos V., Kim R., Freitag M. & Avouris P. Cooling of photoexcited carriers in graphene by internal and substrate phonons. Phys. Rev. B 86, 045413 (2012).

[b41] Graham M. W., Shi S.-F., Ralph D. C., Park J. & McEuen P. L. Photocurrent measurements of supercollision cooling in graphene. Nat Phys 9, 103–108 (2013).

[b42] Betz A. C. *et al.* Supercollision cooling in undoped graphene. Nat Phys 9, 109–112 (2013).

[b43] Song J. C. W., Reizer M. Y. & Levitov L. S. Disorder-Assisted Electron-Phonon Scattering and Cooling Pathways in Graphene. Phys. Rev. Lett. 109, 106602 (2012). 23005313 10.1103/PhysRevLett.109.106602

[b44] Erhard N. *et al.* Ultrafast Photodetection in the Quantum Wells of Single AlGaAs/GaAs-Based Nanowires. Nano Lett. 15, 6869–6874 (2015). 26356189 10.1021/acs.nanolett.5b02766

[b45] Liu C.-H. *et al.* Ultrafast Lateral Photo-Dember Effect in Graphene Induced by Nonequilibrium Hot Carrier Dynamics. Nano Lett. 15, 4234–4239 (2015). 25993273 10.1021/acs.nanolett.5b01912

[b46] Mak K. F., Lui C. H. & Heinz T. F. Measurement of the thermal conductance of the graphene/SiO2 interface. Applied Physics Letters 97, 221904 (2010).

