# Supplementary material for: THz-circuits driven by photo-thermoelectric, gate-tunable graphene-junctions
Source: Sci Rep. 2016 Oct 20;6:35654. doi: 10.1038/srep35654 (PMC5071831; doi:10.1038/srep35654)
Supplement: Supplementary Information [file srep35654-s1.pdf]

Supplementing Information

THz-circuits driven by photo-thermoelectric, gate-tunable graphene-junctions

Andreas Brenneis[1,2], Felix Schade[1,2], Simon Drieschner[1,2], Florian Heimbach[2,3], Helmut Karl[4], Jose A. Garrido[1,2], and Alexander W. Holleitner[1,2]*

[1] Walter Schottky Institut and Physics Department, Technical University Munich, Am Coulombwall 4a, 85748 Garching, Germany.

[2] Nanosystems Initiative Munich (NIM), Schellingstr. 4, 80799 Munich, Germany.

[3] Lehrstuhl für Physik funktionaler Schichtsysteme, Physics Department, Technical University of Munich, D-85748 Garching, Germany

[4] Institute of Physics, University of Augsburg, 86135 Augsburg, Germany.

*corresponding author: holleitner@wsi.tum.de

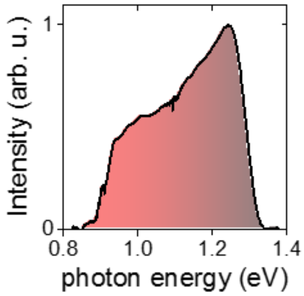

Figure S1: Spectra of the pump laser pulse that is used to excite the graphene at the junction between both gates.

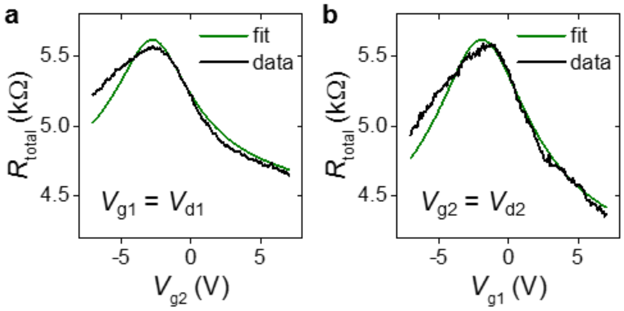

Figure S2: Fit of $R_{\mathrm{total}}(V_{\mathrm{g1}}, V_{\mathrm{g2}})$ for (a) charge neutrality at gate1 ($V_{\mathrm{g1}} = V_{\mathrm{d1}} = -1.9V$) and (b) charge neutrality at gate2 ($V_{\mathrm{g2}} = V_{\mathrm{d2}} = -2.8V$).

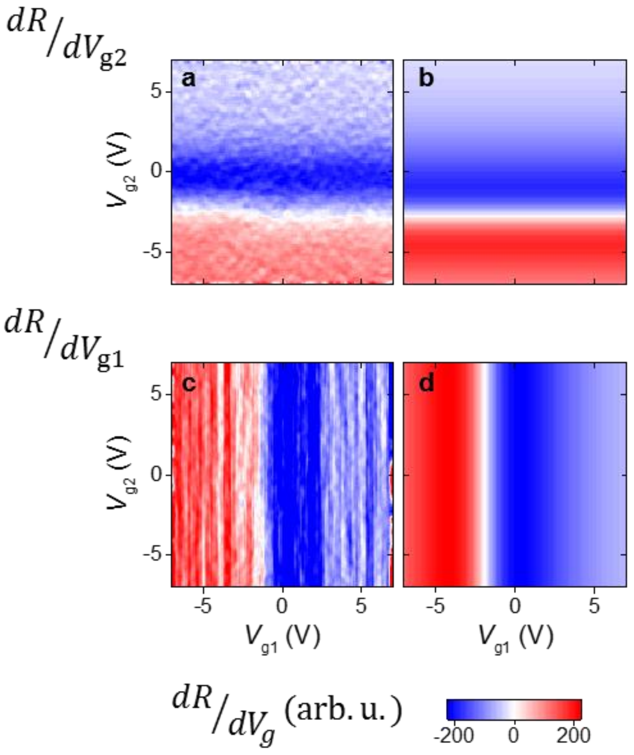

Figure S3: Calculation of the thermopower from the derivative of the resistance. We fit $R_{\text{total}}(V_{\text{g1}}, V_{\text{g2}})$ for $V_{\text{g1,2}} \geq -4$ V to more precisely account for the feature that will determine the manifold sign change of the thermoelectric current for $V_{\text{g1,2}} \geq 0$ V. Generally, a numerical evaluation of the derivative $\frac{\partial R_{\text{total}}}{\partial V_g}$ results in a noisy computed Seebeck coefficient. Figure S5a and S5c depict the numerical results of $\frac{\partial R_{\text{total}}}{\partial V_{g2}}$ and $\frac{\partial R_{\text{total}}}{\partial V_{g1}}$ based on the experimental R_{total}, respectively. For comparison, Figures S5b and S5d depicts $\frac{\partial R_{\text{total}}}{\partial V_{g2}}$ and $\frac{\partial R_{\text{total}}}{\partial V_{g1}}$ that are calculated from the fit of $R_{\text{total}}(V_{\text{g1}}, V_{\text{g2}})$. The charge neutrality point is identical for both methods. Therefore, $R_{\text{total}}(V_{\text{g1}}, V_{\text{g2}})$ is fitted, and we use the resulting parameters to model the thermoelectric current according to equation eq.(3) of the main manuscript.

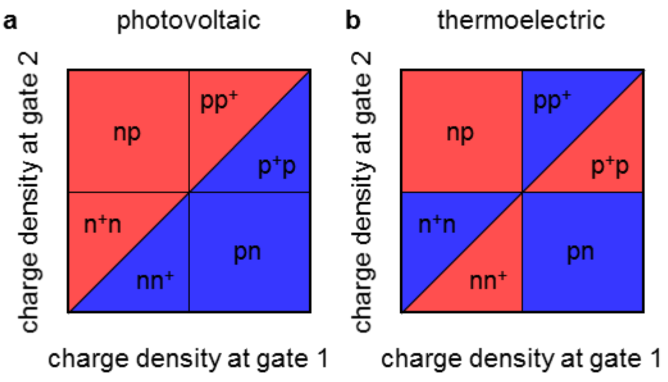

Figure S4: Sketch of the dependence of (a) the photovoltaic and (b) the thermoelectric photocurrent on the charge density in two adjacent graphene regions (red: positive current, blue: negative current).[(1)]

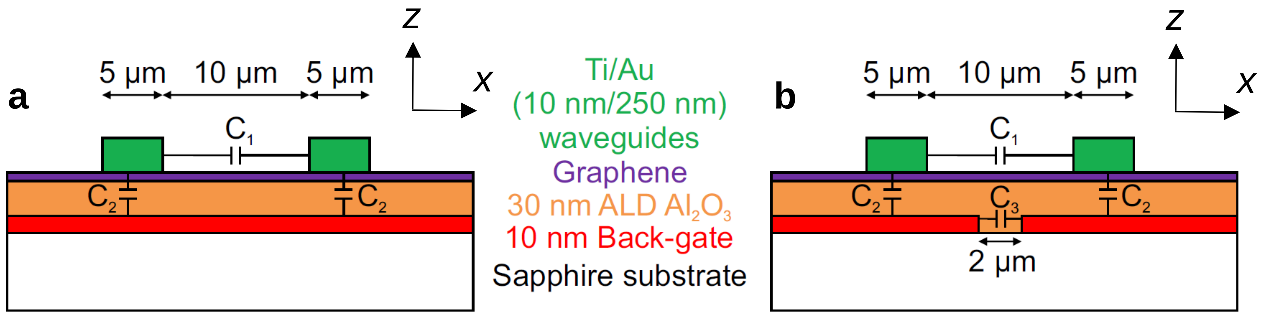

Figure S5: (a) Cross section of a global back-gate below the graphene. In this case, the back-gate spans below both striplines. (b) Sketch of a split back-gate geometry with a lateral distance of 2 µm between the two individual gates. C_1 is the capacitance between the two waveguides and C_2 between one stripline and the back-gate neglecting the impact of graphene. C_3 is the additional capacitance between the two gates of the split back-gate.

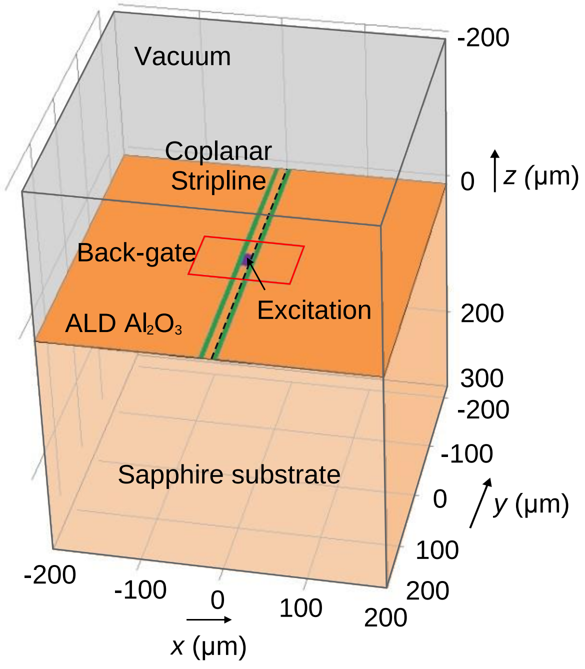

Figure S6: Geometry for the finite element simulations in the time domain using the radio frequency package of Comsol. The 10 nm thin back-gate below the 30 nm thick ALD Al_2O_3-layer has a size of 120 µm times 80 µm. It is indicated by the red rectangle. Both coplanar striplines have a width of 5 µm. They are separated by a distance of 10 µm. The optical excitation of the graphene is simulated by a surface current element between the two waveguides. It has a length of 25 µm in the *y*-direction. For the evaluation of the resulting electromagnetic pulse, the dashed line along the inner side of the right waveguide is used.

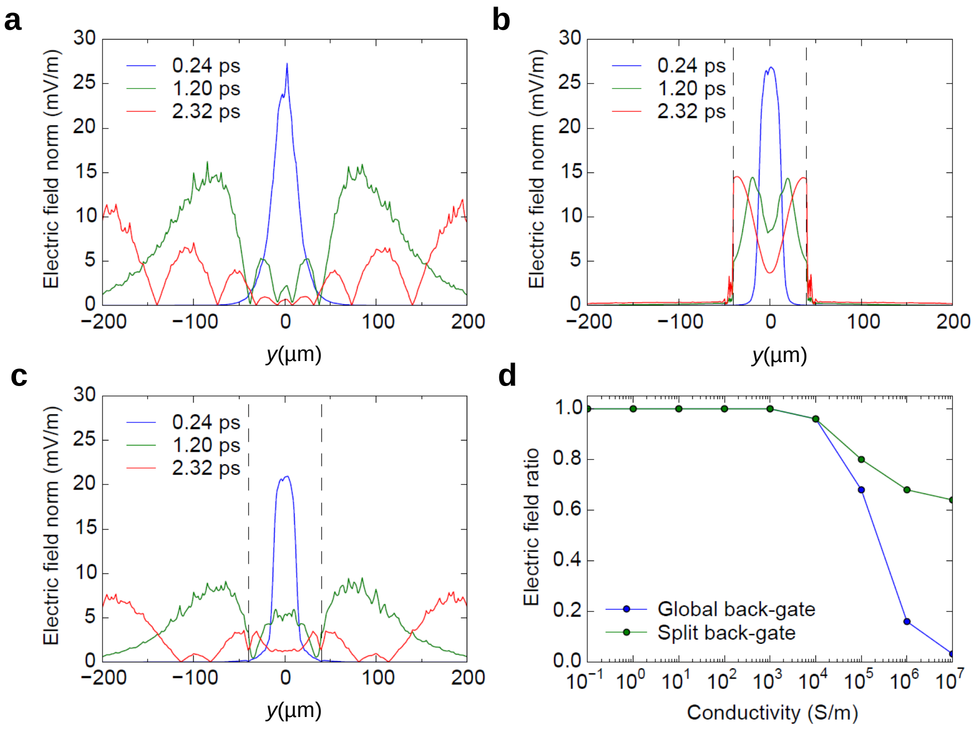

Figure S7: Simulation results for the temporal evolution of an ultrafast current pulse which couples into the coplanar striplines. (a) without a back-gate, (b) with a global metal back-gate (cf. supplementary Figures S5a), and (c) with a metal split back-gate (cf. supplementary Figure S5b). The absolute value of the electric field (norm $E = |\boldsymbol{E}|$) is plotted versus the y-position along the dashed line in Figure S6. The simulations in (b) and (c) are performed using $\varepsilon_r = 3$ and $\sigma = 10^7$ Sm^{-1} for the gate material. The dashed lines in (b) and (c) indicate the extension of the back-gate. (d) Electric field ratio of the pulse amplitude at $y = 200$ µm for $\Delta t = 2.32$ps with a global back-gate (split back-gate) compared to the case without a gate vs. the gate conductivity.

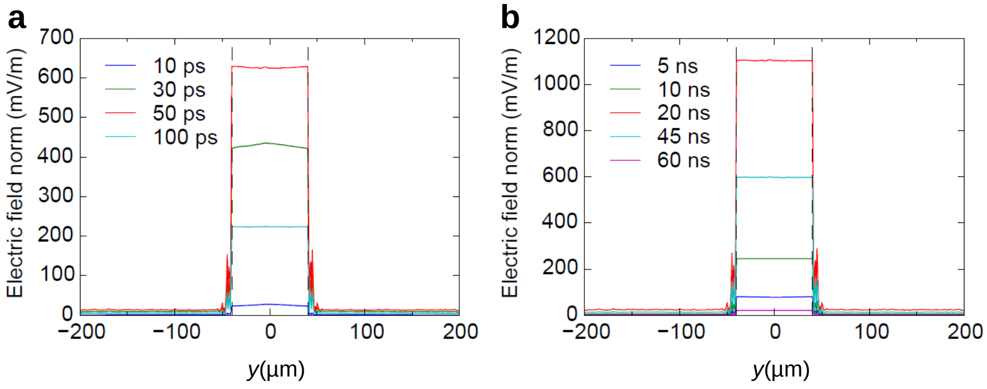

Figure S8: Simulation results for different standard deviations b of the surface current described by supplementary Equation (S3). (a) and (b): $b = 10$ ps (b =10 ns) with a simulation time of 100 ps (100 ns) and time steps of 1 ps (1 ns). The maximum pulse amplitude at the model boundaries accounts for 11 mVm^{-1} in (a) and for 20 mVm^{-1} in (b). The dashed lines indicate the extension of the back-gate.

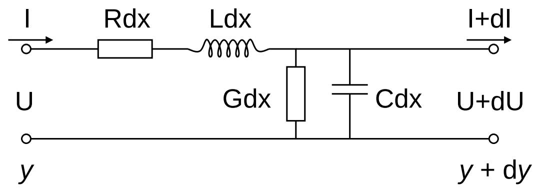

Figure 9: Elementary segment of a transmission line in the y-direction with the infinitesimal change dU (dI) of the voltage U (current I) at $y + dy$, the resistance R, the inductance L, the conductance G and the capacitance C per unit length with the voltage (current) at position y.

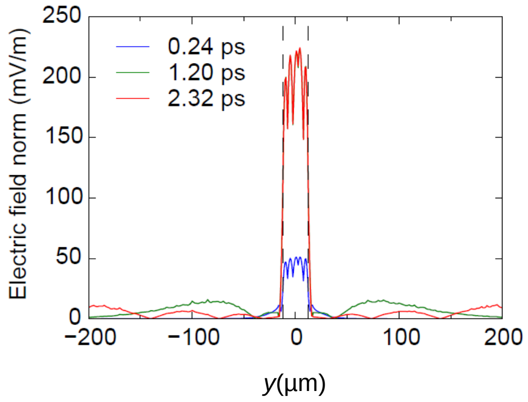

Figure S10: Simulation result when the surface current element is placed below the ALD-grown Al_2O_3-layer and therefore, on top of the back-gate. A pulse propagates along the striplines, because the back-gate capacitively couples into the coplanar stripline. The dashed lines indicate the extension of the surface current element. Since the surface current cannot leave the model during the simulation time, a high absolute value of the electric field arises along the corresponding waveguide position. The maximum pulse amplitude at the model boundaries accounts for 12 mVm^{-1}.

Table S1: Simulation parameters. The relative permittivity ε_r of Al_2O_3 is taken as a mean value from (2)(3) and it is used for both the ALD layer and sapphire substrate. For the back-gate, ε_r varies between the given values for common metals (4)(5)(6). The conductivity for Au waveguides is taken from (7) at room temperature. Furthermore, all materials are supposed to be non-magnetic ($\mu_r = 1$).

	ε_r	μ_r	σ (S/m)
Vacuum	1	1	0
Striplines	1	1	$4.5 \cdot 10^7$ but modeled as ideal conductor
Al_2O_3	9.4	1	0
Back-gate	1 to 200	1	10^{-1} to 10^7 on a logarithmic scale

Supplementary Note: High-frequency electrodynamics of dual-gate junctions integrated in coplanar striplines

In this supplementary note, we present numerical calculations how a dual-gated graphene junction needs to be designed such that high-frequency photo-thermoelectric pulses from a graphene-junction can couple into on-chip THz-striplines. As is demonstrated below, the crucial point is the capacitive interaction of the back-gates with the striplines. To start with, a sketch of the cross section of the sample with a global back-gate below the graphene is shown in the supplementary Figure S5a. The split-gate-geometry used in the ultrafast experiments of the main manuscript is depicted in the supplementary Figure S5b. In both cases, we examine the influence of the back-gate onto an electromagnetic pulse propagating along the coplanar stripline. In particular, we examine a simplified 3-dimensional model of the sample geometry (cf. supplementary Figure S6), which is implemented in the finite element software Comsol Multiphysics. The simulations are performed in the time domain using the so-called radio frequency physics model package (8)(9). Comsol solves the relevant partial differential equation for the vector potential $\boldsymbol{A}$ (S1)

$$\nabla \times \mu_r^{-1}(\nabla \times \mathbf{A}) + \mu_0 \sigma \frac{\partial \mathbf{A}}{\partial t} + \mu_0 \frac{\partial}{\partial t}\left(\varepsilon_0 \varepsilon_r \frac{\partial \mathbf{A}}{\partial t}\right) = 0$$

with ε_r the relative permittivity, μ_r the relative permeability, σ the conductivity, $\varepsilon_0 = 8.85 \cdot 10^{-12}$ Fm^{-1} the vacuum permittivity, and $\mu_0 = 4\pi \cdot 10^{-7}$ Hm^{-1} the vacuum permeability. The electric field $\boldsymbol{E}$ is calculated as (S2)

$$\mathbf{E} = -\nabla V - \frac{\partial \mathbf{A}}{\partial t}$$

with V the electric potential. The wave-equation [supplementary (S1)] as well as equation [supplementary (S2)] are implemented in the Comsol-physics model and can be deduced from the Maxwell equations. The 3-dimensional model (cf. supplementary Figure S6) contains the sapphire substrate, the back-gate, the ALD-grown Al_2O_3-layer, two striplines and a vacuum section on top (all presented measurements in the main manuscript are performed in a vacuum chamber at room temperature). For simplicity, all materials are supposed to be isotropic and linear. The used values for ε_r, μ_r and σ are listed in the supplementary Table S1. All boundaries are modeled as so-called “Surface scattering boundaries”, i.e. every (plane) wave reaching the boundary leaves the model without being reflected or scattered. Such boundaries allow to restrict the model around the relevant area close to the coplanar striplines near the back-gate. The striplines in the supplementary Figure S6 are “planar” and “perfect ideal conductors” ($\boldsymbol{n} \times \boldsymbol{E} = 0$ with the surface normal vector $\boldsymbol{n}$). This choice reduces the computation time and provides the same qualitative simulation results as using realistic modeled striplines (8). The optical excitation of the graphene by the pump laser results in a photo-thermoelectric current. This current couples into the coplanar striplines and it is modeled as a time-dependent “surface current”. The surface current $I(t)$ is assumed to flow in the x-direction and to exhibit a Gaussian shape (S3)

$$I(t) = 10^{-4}\,\mathrm{A\,m^{-1}} \cdot \exp\left(-\frac{(t-T_0)^2}{2b^2}\right)$$

with $b = 0.1$ ps and $T_0 = 3 \cdot$ b. The amplitude of $I(t)$ is similar to the observed ones in the time-resolved measurements. The standard deviation b is chosen to examine the influence of the back-gate on the fast components of the electromagnetic pulse. Furthermore, the overall simulation time is set to be 4 ps with time steps of 0.08 ps. The chosen simulation time exceeds the time which is needed by the pulse to entirely pass through the model boundaries.

In a first reference simulation, the back-gate is omitted and replaced by sapphire. Supplementary Figure S7a shows the pulse propagating along the y-direction (cf. for the dashed line in Supplementary Figure S6) for three different simulated times. At around 0.24 ps ($t \approx T_0$), the surface current $I(t)$ is reaching its maximum. This situation results in the highest electric field norm of the pulse, which couples into the stripline at $y = 0$ µm. At 1.2 ps, a propagating pulse is visible which reaches the model boundaries ($y = -200$ µm and $y = 200$ µm) at around 2.32 ps with an amplitude of $E_{\text{without back-gate}} \approx 1.3 \cdot 10^{-2}$ Vm^{-1}. The additional smaller artifacts are caused by reflections of the surface current between the two striplines. Moreover, the noise of the pulses stems from the minimum mesh size of 1 µm. This size is chosen to minimize the computation time as well as the RAM usage during the simulations. According to (8), a mesh size of 1 µm is sufficient since all occurring frequencies f in the electromagnetic pulse up to the terahertz regime have a wavelength λ at least three times larger. For instance, a frequency of $f = 1$ THz corresponds to a wavelength of $\lambda = c/(n_{\text{eff}} \cdot f) \approx 100$ µm with $n_{\text{eff}} = 2.3$.

In a next simulation, a single back-gate is assumed which spans from one stripline to the other (cf. Supplementary Figure S7b), and which exhibits a metallic conductivity ($\sigma = 10^7$ Sm^{-1}). The simulation shows that no pulse reaches the model boundaries (i.e. the pulse has an amplitude of only $E_{\text{with back-gate}} \approx 4 \cdot 10^{-4}$ Vm^{-1}). Instead, the pulse is reflected in-between the back-gate boundaries in the y-direction. To further analyze this issue, the back-gate conductivity σ is varied as listed in Table S1 using a parametric sweep in Comsol. For each simulation, the ratio of $E_{\text{with back-gate}}/E_{\text{without back-gate}}$ (at $y = 200$ µm for $t = 2.32$ ps) is plotted versus the corresponding conductivity (supplementary Figure S7d). Starting at $\sigma \approx 10^3$ Sm^{-1}, the pulse damping is increased with a higher back-gate conductivity instead of propagating further along the coplanar stripline. The influence of the backgate's relative permittivity ε_r on the simulation results is examined by varying ε_r. Values for ε_r in the range of common metals are used as listed in the supplementary Table S1 for a fixed conductivity. Only a small variation of the pulse amplitudes is found, but no change in the overall temporal evolution. Also increasing the standard deviation b to 10 ps or 10 ns of the surface current (cf. supplementary Equation S3) and adjusting the simulation time gives the same result concerning the damping through the back-gate (cf. supplementary Figure S8). Only on a nanosecond timescale, the pulse amplitude outside the back-gate area rises. The latter explains why the stripline-circuits can be used also for a time-integrated measurement of the photocurrent (I_{photo} in the main manuscript). However, supplementary Figure S7b clearly reveals that time-resolved measurements on a picosecond timescale are not possible using a single metal back-gate which spans from one stripline to the other ($\sigma = 10^7$ Sm^{-1}).

The above simulated results can be further explained by describing the coplanar stripline with the well-known telegraph equations. By solving these differential equations for the elementary segment shown in supplementary Figure S9, a frequency dependent wave impedance of (S4)

$$Z(\omega) = \sqrt{\frac{R + i\omega L}{G + i\omega C}}$$

can be derived (10). The impedance $Z(\omega)$ depends on the resistance R, the inductance L, the conductance G, and the capacitance C per unit length as well as the angular frequency $\omega = 2\pi \cdot f$. Assuming an ideal stripline ($R = 0$, $G = 0$), supplementary equation (S4) simplifies to the frequency independent impedance $Z = \sqrt{L/C}$. The capacitance per unit length is calculated to be $C = \varepsilon_r \varepsilon_0 \cdot l / d$. For simplicity, one can assume that all capacitances can be calculated using the formula of a plate capacitor. For the global back-gate geometry (cf. supplementary Figure S5a), the capacitance between the striplines can be computed to be in the order of $C_1 \approx 0.22$ pFm^{-1} ($\varepsilon_r = 1$ for vacuum, $l = 250$ nm, $d = 10$ µm). The additional capacitance caused by the back-gate C_{gate} is a series connection of two times the capacitance between one stripline and the backgate $C_2 \approx 0.14$ nFm^{-1} ($\varepsilon_r = 9.4$ for ALD-grown Al_2O_3, $l = 5$ µm, $d = 30$ nm, and graphene is neglected). Therefore, the overall capacitance of the coplanar stripline and the back-gate C_{tot} is the sum (parallel connection) of C_1 and $C_{gate} = C_2/2$ resulting in $C_{tot} \approx 0.07$ nFm^{-1}. Thus, the coplanar striplines have a lower impedance at the position of the global back-gate ($Z \propto 1/\sqrt{C_{tot}}$) than without a back-gate ($Z \propto 1/\sqrt{C_1}$) since $C_{tot} >> C_1$. The impedance mismatch at the boundaries of the metal back-gate explains the reflections of the pulse in the y-direction for the case when the back-gate spans from one stripline to the other (supplementary Figure S7b). To reduce the impedance mismatch at the back-gate boundaries along the striplines, C_{tot} should be comparable to C_1. This can be reached by reducing C_{gate}. For this purpose, the back-gate is split in the middle of the striplines into two gates with a distance of 2 µm (supplementary Figure S5b). The new capacitance between the two gates can be estimated to be $C_3 = 0.42$ pFm^{-1} ($\varepsilon_r = 9.4$, $l = 10$ nm, $d = 2$ µm). Now, C_{gate} consists of a series connection of C_3 and two times C_2 resulting in $C_{gate} = (C_2C_3)/(2C_3 + C_2) \approx C_3$, which is much smaller compared to the case of a global back-gate.

All performed simulations for the global back-gate are repeated using the new split back-gate sample geometry. Supplemenatry Figure 7c shows the corresponding temporal evolution of the pulse at three different times in the case of the mentioned split-gate geometry ($\sigma = 10^7$ Sm^{-1}) (cf. supplementary Figure S5a). Now, a pulse reaches the model boundaries. Compared to the simulation without a gate (cf. supplementary Figure 7a), the outcoupling pulse has a lower amplitude. This difference is still caused by the gate boundaries in the y-direction, since C_{gate} is unequal zero. The ratio of $E_{\text{with split back-gate}}/E_{\text{without gate}}$ for the parametric sweep of the gate conductivity σ is shown in supplementary Figure 7d. An apparent result of the geometric adjustment of the sample geometry is an increased electric field ratio of about 62% for $\sigma = 10^7$ Sm^{-1} using a metal split back-gate. Hence, a metal split-gate is suitable for allowing time-resolved measurements on photo-thermoelectric graphene-junctions.

We note that instead of a geometric adjustment, a suitable global back-gate material with a conductivity σ below 10^3 Sm^{-1} might appear equally suitable (cf. supplementary Figure S7d). In a naïve picture, this can be understood that reducing the conductivity is equivalent to removing an electrode with a capacitance C_{gate}. We note that the utilized O^+-ion-implanted silicon on the sapphire substrate has a conductivity of approx. $\sigma = 10^{-1}$ Sm^{-1}. In this sense, in principle, it could be used as a gate material. However, it has a bandgap which is smaller than the pump laser energy. Therefore, the back-gate would be photoactive. To simulate the impact of such a scenario, the surface current element is placed below the ALD-grown Al_2O_3-layer (see Comsol simulation in the supplementary Figure S10). The additionally generated photocurrent capacitively couples from the photo-active back-gate into the coplanar striplines. Hence, the photoresponse of graphene and a possible silicon back-gate would overlay in time-resolved measurements. Another issue with a low conductive gate is that as in the case of O^+-ion-implanted silicon, such materials typically have a strongly temperature-dependent conductivity. In other words, cooling the sample with liquid nitrogen or even helium freezes-out the charge carriers and therefore, turns the conductivity towards zero. In turn, a gating of the graphene would no longer be possible.

To summarize, in this supplementary note, we present a design guideline for a split back-gate geometry that is suitable for time-resolved measurements on dual-gated graphene-junctions and in a broader sense, for the integration of photo-thermoelectric graphene-junctions into high-frequency circuits.

References:

(1) J.C. SONG, M.S. RUDNER, C.M. MARCUS, L.S. LEVITOV, "Hot Carrier Transport and Photocurrent Response in Graphene". Nano Lett. 11, 4688–4692 (2011).

(2) W. B. COOK and S. PERKOWITZ, "Temperature dependence of the far-infrared ordinary ray optical constants of sapphire", Applied Optics 24, 1773 (1985).

(3) K. Z. RAJAB, M. NAFTALY, E. H. LINFIELD, J. C. NINO, D. ARENAS, D. TANNER, R. ITTRA, and M. LANAGAN, "Broadband dielectric characterization of aluminum oxide (Al2O3)", J. Micro. and Elect. Pack. 5, 101–106 (2008).

(4) M. POLYANSKIY, RefractiveIndex.INFO, http://refractiveindex.info/

(5) M. A. ORDAL, R. J. BELL, R. W. ALEXANDER, L. L. LONG, and M. R. QUERRY, "Optical properties of fourteen metals in the infrared and far infrared: Al, Co, Cu, Au, Fe, Pb, Mo, Ni, Pd, Pt, Ag, Ti, V, and W", Applied Optics 24, 4493–4499 (1985).

(6) A. D. RAKI´C, A. B. DJURIŠI´C , J. M. ELAZAR, and M. L. MAJEWSKI, "Optical properties of metallic films for vertical-cavity optoelectronic devices", Applied Optics 37, 5271–5283 (1998).

(7) R. A. MATULA, "Electrical resistivity of copper, gold, palladium, and silver", Journal of Physical and Chemical Reference Data 8, 1147–1298 (1979).

(8) COMSOL, RF Module, https://www.comsol.com/rf-module

(9) O. C. ZIENKIEWICZ, R. L. TAYLOR, and J. Z. ZHU, The Finite Element Method: Its Basis and Fundamentals, 7th ed. (Butterworth Heinemann, 2013)

(10) J. D. JACKSON, Classical Electrodynamics, 3rd ed. (Wiley, 1998).
